# Public health facility quality and child immunization outcomes in rural India: A decomposition analysis

**DOI:** 10.1016/j.vaccine.2022.03.017

**Published:** 2022-04-06

**Authors:** Amit Summan, Arindam Nandi, Emily Schueller, Ramanan Laxminarayan

**Affiliations:** a Center for Disease Dynamics, Economics & Policy, Washington DC, USA; b The Population Council, 1 Dag Hammarskjold Plaza, New York, NY 10017, USA; c High Meadows Environmental Institute, Princeton University, NJ, USA; d University of Maryland, MD, USA

**Keywords:** India, Routine immunization, UIP, INCHIS, Determinants

## Abstract

Universal coverage of routine childhood vaccines remains a challenge in many low- and middle-income countries (LMICs). In India, vaccination campaigns have increased full immunization coverage among 12–23 month old children from an estimated 62% in 2015–2016 to 76% in 2019–2020. Long-term improvements in coverage will likely require systemic changes to both the supply and demand sides of immunization programs. However, the effect of health system inputs on child vaccination outcomes remains poorly quantified in India. We examined the association between the quality of public health facilities and child vaccination outcomes in rural India using data from the nationally representative Integrated Child Health and Immunization Survey (2015–2016) which covered 1,346 public primary health sub-centers and 44,571 households. We constructed two indices of sub-center quality using multiple correspondence analysis: one related to the general health infrastructure quality and the other measuring vaccine service delivery. Using probit regression, we analyzed the relationship between vaccination outcomes in children under 2 years of age and sub-center quality, controlling for household socioeconomic characteristics. Additionally, we conducted Fairlie decomposition analysis by wealth group — bottom wealth quintile relative to the top four wealth quintiles— to examine factors contributing to gaps in immunization between rich and poor households. Infrastructure quality index was positively associated with completion of seven vaccination outcomes: full immunization, DPT-1 (first dose of diphtheria, pertussis, and tetanus), DPT-2, DPT-3, Bacillus Calmette–Guérin (BCG), hepatitis B (birth dose), and on-time vaccination (OTV). Vaccine service delivery index was positively associated with completion of measles vaccination. The distribution of infrastructure quality contributed to increased gaps in full immunization and OTV between rich and poor households, while greater proximity to vaccination site for poorer households reduced these gaps. Improved quality of health facilities, particularly facilities used by low-income households, may improve vaccination outcomes.

## 1 Introduction

Despite significant spending on childhood immunization, many low-and middle-income countries (LMICs) have not achieved universal coverage of routine childhood vaccines. In India, vaccine-preventable diseases such as pneumonia, diarrheal diseases, measles, and meningitis accounted for>400,000 under-five deaths in 2015 alone [1]. Understanding the obstacles to vaccination at a local level is crucial for improving child mortality and health outcomes in India and other LMICs. Important demand-side determinants of child vaccination coverage in LMICs include household income and social status, parental knowledge, and religious and cultural beliefs [2], [3], [4], [5], [6], [7], [8], [9], [10]. In terms of supply-side factors, distance to immunization session site, low quality of services, and lack of facility resources are known reasons for non-vaccination of children [11], [12]. Recent policy efforts in India such as Mission Indradhanush, an effort to use a campaign mode to increase routine immunization coverage in India have increased full immunization coverage to an estimated 76% [13], [14], but gaps in coverage driven by both supply-side and demand-side factors persist. Rural areas of India — where 94% of children received the majority of vaccines in public health facilities — continue to lag urban areas for all vaccines [15].

While parents report poor quality of healthcare facilities as an important deterrent to child vaccination, healthcare quality itself – and its association with vaccination coverage – remains inadequately quantified in LMICs. Previous literature has instead focused mainly on access to healthcare. For example, a recent study in India found that the availability of health center (primary health sub-center [SC] or primary health center [PHC]) or health care workers (auxiliary nurse midwives or accredited social health activists) did not significantly reduce Diphtheria, Pertussis, Tetanus (DPT) vaccination dropout rates [16]. Another study found that rural Indian children who had a hospital within 2 km of their village were 4.8% less likely to miss non-polio vaccine doses [17]. The authors found no association between the availability of a community health worker in the village and vaccination rates. A third study focused on the availability of health facility near households within the slums of Agra, India and identified a positive association with vaccination coverage [18]. In the Indian state of West Bengal, availability of health workers and equipment at SCs was found to have a positive association with month-specific vaccine coverage [19]. A study in Burkina Faso found no associations between the availability of physical and human resources at the health facility serving a community and the community’s vaccination coverage [20].

Previous studies [16], [17], [19] have primarily focused on access to healthcare rather than the quality of healthcare. Three of the four India-focused studies referenced above looked at the availability of a public health facility or health worker within a household’s village or district but did not account for the quality of those facilities or workers [16], [17], [18], while a fourth which focused on a state within India included very limited measures of quality [19]. In LMICs, the quality of care is a better predictor of health outcomes than access to healthcare facilities [21], [22]. In 2016, an estimated five million excess deaths in LMICs were attributable to poor quality of health care alone [21].

Measurable indicators of quality that are related to vaccination coverage rates can inform policies for immunization program funding in India and other LMICs. We examined the socioeconomic and healthcare quality determinants of coverage rates and timeliness of routine child immunization in rural India. We also conducted in-depth analyses of the distribution of facility quality and its association with vaccination outcomes across income groups using decomposition methods.

## 2 Data and methods

### 2.1 Data

We used data from India’s Integrated Child Health and Immunization Survey (INCHIS), a nationally representative, stratified, cross-sectional household survey conducted over three two-month rounds between March 2015 and April 2016 [23]. INCHIS collected data on vaccination outcomes and access to public health facilities for children below the age of 24 months, and the quality of health facilities at the village level. It covered 44,571 households and 1,346 primary health SCs in 24 states. The first, second, and third rounds covered 11,683, 15,039, and 17,849 households, and 402, 436, and 508 SCs, in 83, 81, and 96 districts, respectively. Each INCHIS round collected data from 12 states; six states— Bihar, Maharashtra, Madhya Pradesh, Rajasthan, Telangana, and Uttar Pradesh — were fixed for every round and six states were rotated each survey round. States were selected to ensure representation from each region and income level. INCHIS employed three-stage stratified sampling design within each state where district, cluster (village/urban ward), and households were selected at three different stages.

We matched each household to its nearest SC. While SCs were primarily located in rural areas, some *peri*-urban households were reported as being served by SCs. Immunization status information of the youngest child in the household was collected from vaccination cards and through caregiver recall when cards were unavailable.

We analyzed the relationship between eight binary vaccination outcomes of children and health facility characteristics, controlling for socioeconomic indicators. Vaccination outcomes included: DPT-1 (first dose of diphtheria, pertussis, and tetanus), DPT-2, DPT-3, first dose of measles, hepatitis B given at birth (HepB), Bacillus Calmette–Guérin (BCG), full vaccination, and a measure of on-time vaccination (OTV). Table 1 describes each vaccine and the appropriate age for vaccination, according to the Indian Academy of Pediatrics [24]. We excluded children who were reported as being vaccinated before the eligibility age for a vaccine (0.6% of the sample) to reduce potential measurement errors.

**Table 1:** Description of vaccination indicators.

Vaccine	Definition	Recommended age
BCG	Bacillus Calmette–Guérin	at birth
Hep B0	Hepatitis B	at birth
DPT-1	Provides vaccination against diphtheria, pertussis (whooping cough), and tetanus, and requires three doses and a fourth booster dose.	6 weeks
DPT-2		10 weeks
DPT-3		14 weeks
Measles1	First dose of measles vaccine	9 to 12 months
Fully vaccinated	A child is considered fully vaccinated when they receive one dose of Bacillus Calmette–Guérin, three doses of DPT and polio and one dose of measles. Full immunization can occur in children as early as 9 months of age and is typically evaluated at 12 months of age.	12 months
OTV	On-time vaccination defined as child eligible for DPT-1, DPT-2, DPT-3, and full immunization having been vaccinated 28 days after becoming eligible for the respective vaccine. Child evaluated for last vaccination they were eligible for.	See above

Source: Indian Academy of Pediatrics [24].

A child was considered fully vaccinated if they received one dose of BCG, three doses of DPT and polio, and one dose of the measles vaccine. The OTV indicator examined the appropriate timing of the child’s vaccination. We considered timely vaccination of DPT-1, DPT-2, DPT-3, and full immunization. OTV had a value of 1 if the child had received the respective vaccine within 28 days after the recommended age for vaccination as described in Table 1. This is consistent with previous studies which have considered vaccination to be timely if it was done within 30 days of eligibility [25], [26]. Each child was evaluated for the vaccine they were most recently eligible for resulting in one observation per child. Consider a child who was 19 weeks of age at the time of the survey. The last vaccine for which they were eligible for would be the DPT-3 dose which has an eligibility age of 14 weeks. If the child received the vaccine between 14 and 18 weeks of age, the child would be considered timely immunized for DPT-3 according to our definition. Their value of OTV would be 1. They would not be evaluated for timely vaccination of DPT-1 and DPT-2 under this definition, but only DPT-3 as it was the most recent vaccine for which they were eligible. We evaluated children who were above the age of 12 months for full vaccination; although a child can be fully immunized as early as 9 months —earliest recommended age for the measles vaccine (last vaccine received to be considered fully immunized) — the highest recommended age for measles is 12 months. In additional sensitivity analysis, we also examined the timeliness of receiving all past doses, not just the most recent dose. We constructed a composite *OTV_Cd* variable which had a value of 1 only if all four vaccinations, DPT-1, DPT-2, DPT-3, and full immunization, were received within 28 days of eligibility age, and 0 otherwise. Also a continuous variable varying between 0 and 1, *OTV_C*, was constructed which received an incremental value of 0.25 for each vaccine that was a received on-time.

### 2.2 Measures of quality of care at primary health Sub-centers

SCs are the first contact point with the public health system in rural India. There is one SC mandated for every 5,000 people (or 3,000 people for tribal or remote areas). In 2018, there were 158,417 SCs serving rural India at the rate of approximately 5,600 people per SC [27]. Each SC is staffed with at least one auxiliary nurse midwife (ANM) whose responsibilities include child immunization activities under the UIP [28], [29]. Vaccines are not always administered on-site at SCs; SC workers may be responsible for conducting immunization sessions at outreach facilities such as an *Anganwadi* (maternal and child health and welfare center) or community health events such as the village health and nutrition day [30], [31]. In INCHIS, 68% of child vaccinations were reported to be through SCs or *Anganwadi* (mother–child nutrition and welfare) centers [29].

We employed multiple correspondence analysis (MCA) to construct two indices of the quality of care related to immunization services in SCs – an infrastructure quality index and an immunization service delivery index. MCA is a dimension reduction technique analogous to the commonly employed principal component analysis but for categorical data [32]. It has been applied widely to construct health and asset indices in earlier studies [33], [34], [35]. MCA analyzes the association between groups of variables by transforming all data to a matrix of all two-way cross tabulations across categorical variables (Burt matrix) or to an indicator matrix where possible variable levels are coded as binary variables [36]. The transformed data can be represented in multi-dimensional space where associations between variables are determined by the chi-squared distance across groups of variables and observations [36]. MCA identifies the key dimensions underlying this data; the first dimension can be considered as an unobserved latent variable which captures the greatest variance (known as inertia) from the original variables [37]. The second dimension is orthogonal to the first dimension and contains the second most amount of variance, and so on. We obtained the indicator score for each facility by taking the weighted average of all categorical variables, from weights generated by MCA for the first dimension. Based on the MCA generated index scores, we assigned SCs to a tercile for each index.

The first index measured SC infrastructure quality and resource availability. This index modelled the following indicators: the cleanliness of the facility (good, fair, or poor), availability of a telephone, availability of toilet, the quality and reliability of water source (piped, bore/tube well, hand pump, well, external well, no water supply, or other water supply), and the availability of regular power supply (regular power supply, irregular power supply, regular power supply with power cut in summer, regular power cut, or no electricity). We included the following schooling indicators of village health workers who are known as the accredited social health activists (ASHAs) and tasked with improving child vaccination rates – illiterate, literate but no formal schooling, <8th standard (grade), 8th standard to higher secondary, and graduate or above.

The second index evaluated immunization service delivery from 23 binary variables, each identifying whether the auxiliary nurse midwife (ANM) had experienced a shortage of essential items required for vaccination such as vaccines, syringe, and diluents, along with two basic medications (zinc tablet and paracetamol), in the last six months. Table 2 describes the variables included in the estimation of the indices. All variables used to construct the quality indices and other control variables for analysis, described below, were drawn from INCHIS data.

**Table 2:** Index components.

Index	Infrastructure quality	Vaccine and equipment availability*^+^	
Indicators measured	ASHA education	BCG/ BCG diluent	5 ml reconstitution syringes
	Building type	DPT	Auto disable syringes
	Cleanliness	Hepatitis B	IFA Tablet (adult/kids)
	Electricity source	JE/ JE diluent	MCP Card
	Infrastructure condition	Measles/ Measles diluent	ORS Packet
	Telephone availability*	OPV	Paracetamol
	Toilet availability*	Pentavalent	Plastic spoon/cap
	Water source	TT	Red and black bags
		Zinc tablet/syrup	Vitamin A solution
Total index inputs	8	23	

*Binary variables coded yes and no indicating availability of item. ^+^Shortage in last 6 months.

Note: *ASHA* = Accredited Social Health Activists; *BCG* = Bacillus Calmette–Guérin; *OPV* = Oral Polio Vaccine; *DPT* = Diphtheria, Pertussis, Tetanus; *TT* = Tetanus Toxoid; *JE* = Japanese encephalitis; *MCP* = Mother and Child Protection Card; *ORS* = Oral Rehydration Salt; *IFA* = Iron Folic Acid.

### 2.3 Probit and fractional probit regression analysis

We conducted probit regression analyses to evaluate the associations between each vaccination outcome and sub-center characteristics. The model included indicators of the top two terciles of the two SC quality indices and time taken to reach to immunization facility as reported by households (15 to 30 min, and>30 min). The model covariates also included a set of household and socioeconomic indicators that have been found to affect vaccination outcomes: region (east, north, northeast, south, or west), locality (rural or urban), wealth quintile (top four wealth quintiles), religion (Christian, Muslim, Sikh, or other religion), caste (scheduled caste, schedule tribe, or other backward caste), household size (greater than five), mother’s education level (primary or lower, middle to secondary, and graduate and above), mother’s age, child’s age and gender, and whether the child was born in a health facility. Wealth quintile was constructed using MCA on 18 binary variables measuring ownership of assets [23].

In sensitivity analysis we replaced the main OTV variable with the *OTV_Cd* variable which had a more stricter definition of timeliness as discussed in the previous section. In addition to the probit model, we conducted a fractional probit regression of *OTV_C*, the continuous composite variable evaluating timely vaccination of all vaccines, as a sensitivity analysis to the simple OTV variable, on the above variables. Standard errors were clustered at the district level, and survey weights for the child were applied to account for sampling design. Data were analyzed using STATA version 14.2, and we considered p < 0.05 for statistical significance.

### 2.4 Fairlie decomposition of standard of living

Standard of living - as measured by wealth quintile – is an important determinant of health and access to healthcare in LMICs [5], [29], [38]. Individuals in lower wealth quintiles are more likely to belong to minority caste groups, religious groups, live in rural areas, or have lower schooling levels. Public health facilities are meant to reduce such inequities in access and quality of care however, unequal distribution of public resources may be reflected in unequal distribution of health facility quality across wealth groups, and exacerbate rather than reduce vaccination gaps.

We employed the Fairlie decomposition method [39] to further analyze vaccination outcomes of households in the bottom wealth quintile relative to the top four wealth quintiles. The Fairlie decomposition method is an extension of the Oaxaca-Blinder decomposition method, but for variables with binary outcomes [40], [41]. It has been used to analyze differences in outcomes between groups (e.g., sex or race) for health and labor outcomes [42], [43], [44], including in immunization studies [45], [46]. We decomposed full immunization and timely vaccination differences between the lowest and the top 4 wealth quintile groups. The methodology is briefly described below.

We started with a probit regression model as follows:

(1) $$\mathrm{Pr}\left(I_{i}^{j}=1|X_{i}\right)=\omega \left({X_{i}}^{\text{'}}B^{j}\right)=\frac{e^{X_{i}\text{'}B^{j}}}{1+e^{X_{i}\text{'}B^{j}}}$$

Where ω is the cumulative standard normal distribution function and PR($I_{i}$) indicates the probability that child *i* received vaccine *j*, which is regressed upon the covariate set *X*. Regression coefficients are denoted by *B*.

Following Eq. (1), the difference in vaccination outcomes for rich and poor households can be written as follows:

(2) $$\overset{¯}{I^{R}}-\overset{¯}{I^{P}}=\omega \left({{\overset{¯}{X}}^{R}}^{\text{'}}B^{R}\right)-\omega \left({{\overset{¯}{X}}^{P}}^{\text{'}}B^{P}\right)$$

Where $\overset{¯}{I^{i}}$ is the average probability of being vaccinated for type *i* households*,* ${{\overset{¯}{X}}^{i}}^{\text{'}}$ is a vector of mean values of explanatory covariates for type *i* households and $B^{i}$ is the vector of estimated coefficients for type *i* households. The two types of households considered are rich, *R*, and poor, *P*, households. By adding and subtracting counterfactual immunization outcomes for poor households with a distribution of explanatory variables equivalent to rich households, $\omega \left({{\overset{¯}{X}}^{R}}^{\text{'}}B^{P}\right)$, Eq. (2) can be rewritten as:

(3) $$\overset{¯}{I^{R}}-\overset{¯}{I^{P}}=\left[\omega \left({{\overset{¯}{X}}^{R}}^{\text{'}}B^{R}\right)-\omega \left({{\overset{¯}{X}}^{P}}^{\text{'}}B^{R}\right)\right]+\left[\omega \left({{\overset{¯}{X}}^{R}}^{\text{'}}B^{R}\right)-\omega \left({{\overset{¯}{X}}^{R}}^{\text{'}}B^{P}\right)\right]$$

The Fairlie decomposition method estimates the difference attributable to the two components. The first term in Eq. (3) is the endowment component, which is the explained difference in outcomes due to differences in distribution of the explanatory variables. It measures the difference in predicted probability of immunization after replacing the distribution of explanatory variables in rich households to be equal to those of poorer households. The second term in Eq. (3) is the return individuals or households receive to these endowments. In health systems studies, this latter component can be attributed to structural differences in how health systems benefit different groups [42], [43], [44].

## 3 Results

### 3.1 Summary statistics of the study sample

Table 3 shows vaccination status by background socioeconomic and demographic characteristics and quality of household’s SC. Children in the highest and lowest wealth quintiles had substantial differences in full vaccination rates, 80% and 58%, respectively. Children with mothers who had graduate degrees had 75% coverage of full vaccination as compared with 59% among children whose mothers had no schooling. There was also a large urban–rural divide in vaccination rates — rural children had 80% full vaccination coverage as compared with 66% among urban (*peri*-urban areas) children. Children with access to an SC in the highest tercile for vaccine service delivery and infrastructure quality index had full vaccination rates of 69% and 74%, in comparison with 67% and 63% full vaccination coverage, respectively, for children who had access to an SC in the lowest tercile. Infants from socioeconomically disadvantaged tribal groups (scheduled tribe), Muslim, and Christian households, and those born at home also had lower vaccination rates when compared with the respective reference groups.

**Table 3:** Background characteristics of study children by vaccination status (%).

	**Full**		**DPT-1**		**DPT-2**		**DPT-3**		**Measles**		**BCG**		**Hepatitis B**		**OTV**	
	**No**	**Yes**	**No**	**Yes**	**No**	**Yes**	**No**	**Yes**	**No**	**Yes**	**No**	**Yes**	**No**	**Yes**	**No**	**Yes**
**Vaccine Availability Score**																
1	33	67	14	86	20	80	30	70	21	79	6	94	20	80	39	61
2	34	66	16	84	23	77	35	65	22	78	8	92	21	79	42	58
3	31	69	12	88	19	81	30	70	17	83	5	95	16	84	38	62
**Infrastructure Score**																
1	37	63	18	82	25	75	38	62	24	76	9	91	26	74	46	54
2	30	70	13	87	19	81	30	70	19	81	5	95	17	83	37	63
3	26	74	9	91	14	86	24	76	16	84	3	97	10	90	32	68
**Region**																
Central	35	65	10	90	19	81	33	67	14	86	3	97	11	89	41	59
East	24	76	12	88	16	84	25	75	14	86	3	97	17	83	32	68
North	43	57	21	79	29	71	43	57	29	71	12	88	30	70	51	49
Northeast	41	59	16	84	22	78	33	67	25	75	10	90	32	68	44	56
South	22	78	4	96	8	92	17	83	17	83	1	99	2	98	25	75
West	30	70	13	87	19	81	30	70	20	80	6	94	14	86	36	64
**Sex**																
Male	32	68	14	86	20	80	32	68	20	80	6	94	19	81	39	61
Female	33	67	15	85	21	79	32	68	21	79	6	94	19	81	40	60
**Locality**																
Rural	34	66	15	85	21	79	33	67	21	79	7	93	20	80	41	59
Urban	20	80	8	92	13	87	20	80	15	85	2	98	12	88	27	73
**Wealth Quintile**																
1	42	58	21	79	29	71	41	59	27	73	10	90	28	72	49	51
2	31	69	13	87	20	80	32	68	18	82	6	94	18	82	39	61
3	28	72	10	90	16	84	27	73	18	82	4	96	14	86	34	66
4	25	75	8	92	13	87	23	77	16	84	2	98	10	90	31	69
5	20	80	7	93	12	88	21	79	12	88	2	98	10	90	28	72
**Religion**																
Hindu	31	69	13	87	20	80	31	69	19	81	6	94	18	82	39	61
Muslim	40	60	20	80	27	73	38	62	29	71	10	90	26	74	46	54
Christian	38	62	14	86	20	80	29	71	20	80	7	93	20	80	38	62
Sikh	12	88	5	95	11	89	18	82	6	94	4	96	6	94	22	78
Other	30	70	6	94	14	86	27	73	15	85	3	97	22	78	36	64
**Caste**																
General	28	72	13	87	18	82	28	72	18	82	6	94	18	82	35	65
Scheduled Tribe	39	61	15	85	22	78	36	64	18	82	5	95	17	83	44	56
Other Backward Caste	33	67	15	85	21	79	33	67	22	78	7	93	20	80	40	60
Scheduled Caste	33	67	13	87	21	79	32	68	21	79	6	94	19	81	40	60
**Education**																
No Schooling	41	59	21	79	28	72	40	60	26	74	10	90	27	73	48	52
Primary Or Lower	31	69	12	88	19	81	31	69	18	82	5	95	15	85	38	62
Middle To Secondary	25	75	10	90	15	85	25	75	16	84	4	96	15	85	33	67
Graduate	25	75	6	94	12	88	23	77	14	86	2	98	11	89	33	67
**Household Size**																
< 5	29	71	12	88	18	82	29	71	19	81	5	95	16	84	37	63
> 5	34	66	15	85	21	79	33	67	21	79	7	93	20	80	41	59
**Place of Birth**																
Institutional	29	71	12	88	18	82	29	71	17	83	4	96	14	86	37	63
Non-Institutional	45	55	24	76	32	68	45	55	31	69	14	86	37	63	52	48
**Distance to Vaccination Site**																
< 15 Minutes	30	70	13	87	19	81	30	70	18	82	6	94	19	81	37	63
15 To 30 Minutes	34	66	14	86	21	79	32	68	21	79	6	94	19	81	40	60
> 30 Minutes	36	64	20	80	25	75	35	65	22	78	7	93	20	80	43	57
Observations	11,898		23,863		23,092		22,302		16,259		24,508		24,508		22,139	

Note: Data are from INCHIS 1, 2, and 3 surveys. Numbers are percentages for each vaccination outcome binary indicator e.g., whether fully vaccinated (yes/ no). *Full =* 1 dose BCG and measles, 3 doses of DPT and polio; *HepB* = Hepatitis B given at birth; *DPT* = Diphtheria, Pertussis, Tetanus; *BCG* = Bacillus Calmette–Guérin; *OTV* = On-time vaccination – considers timely vaccination of DPT and full immunization.

**Fig. 1:**
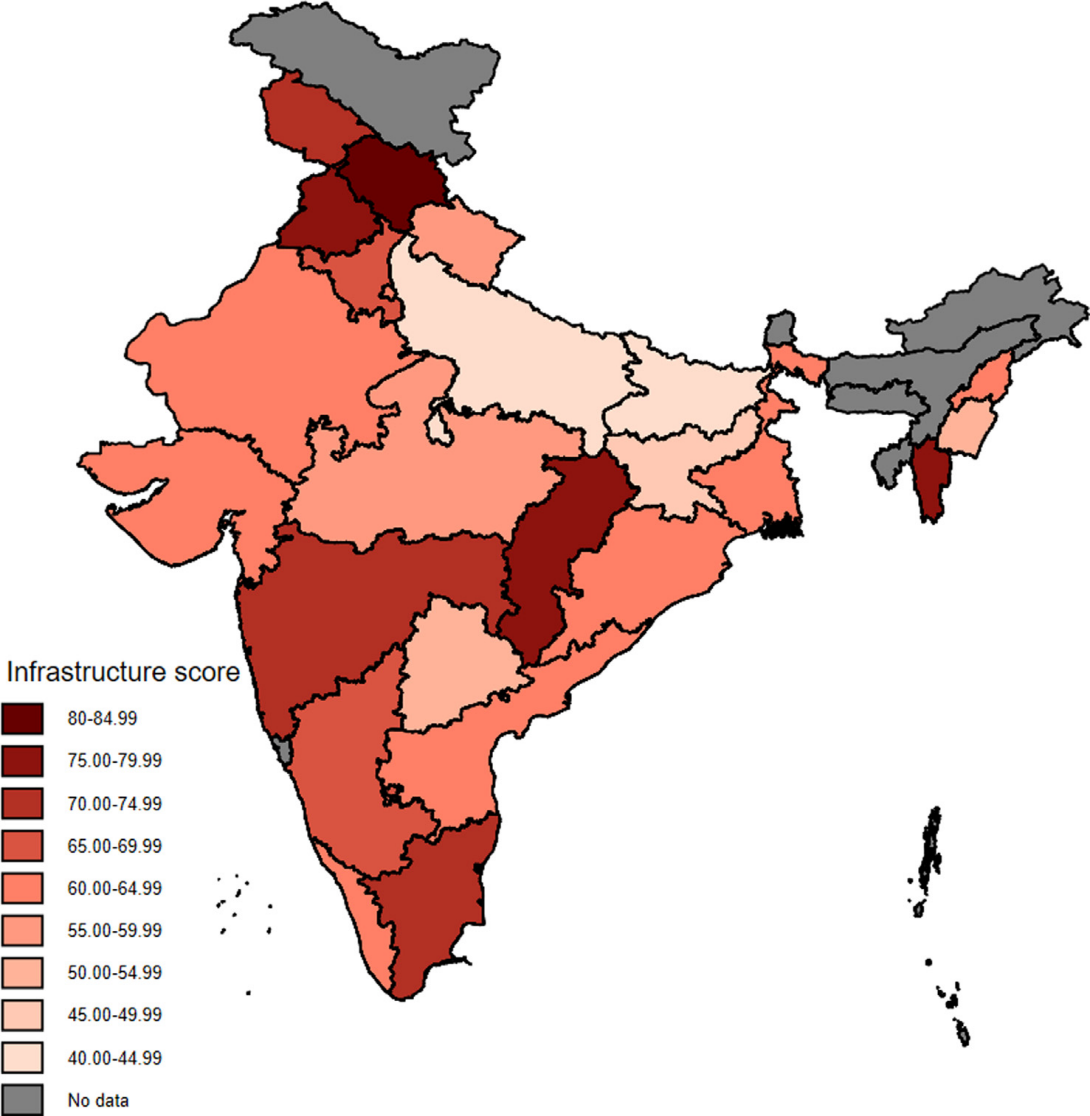
**Infrastructure quality score of sub-centers across states in India.** Note: Map coordinate data are from Database of Global Administrative Areas, version 2.8 (2015). Colors denote the mean state score of infrastructure quality constructed using multiple correspondence analysis, where sub-center scores were standardized to be between 0 and 100. Scores were averaged using state household weights.

**Fig. 2:**
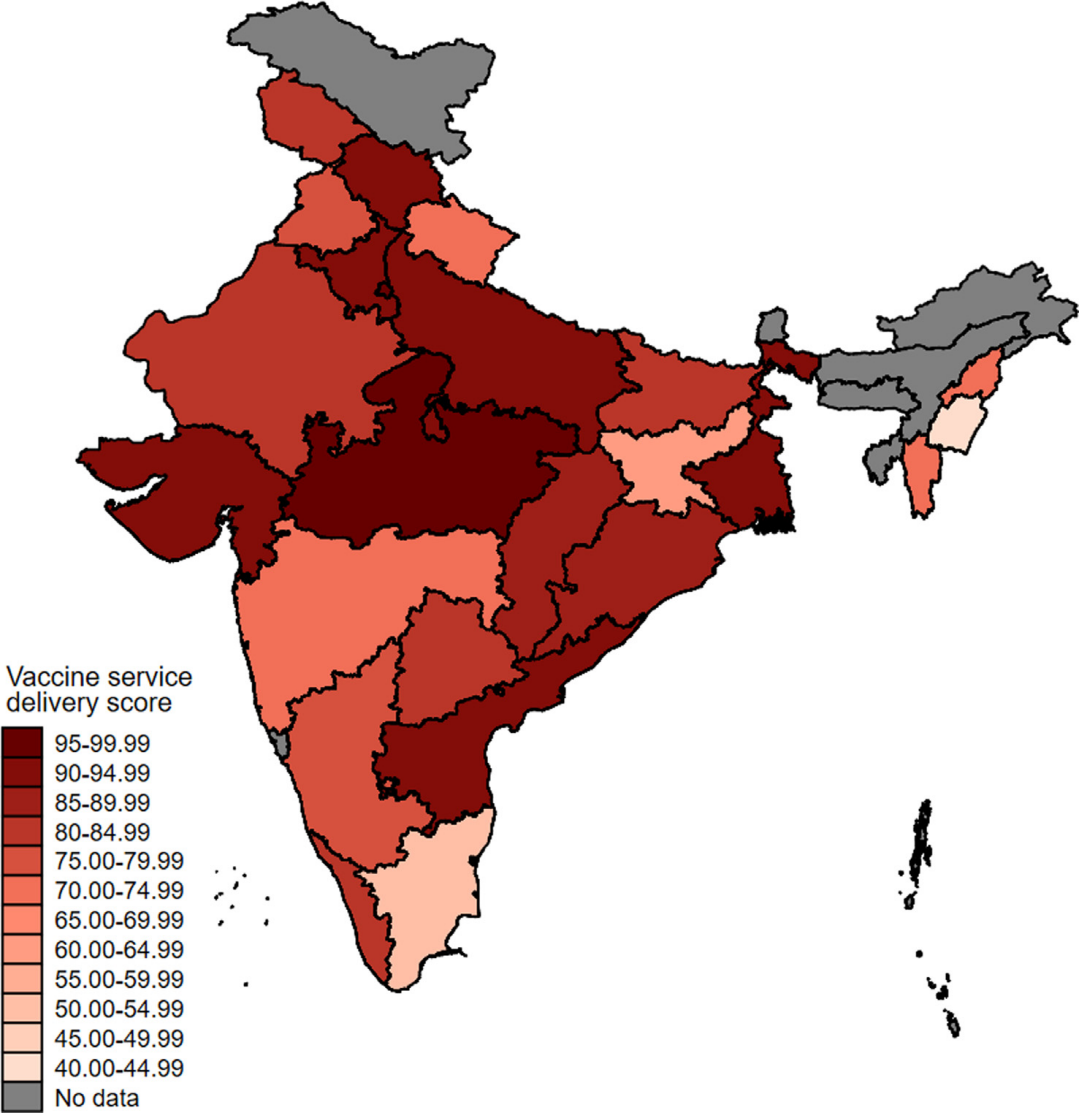
**Vaccine service delivery score of sub-centers across states in India**. Note: Map coordinate data are from Database of Global Administrative Areas, version 2.8 (2015). Colors denote the mean state score of vaccine service delivery score constructed using multiple correspondence analysis, where scores were standardized to be between 0 and 100. Scores were averaged using state household weights.

Fig. 1, Fig. 2 show the distribution of infrastructure quality score and vaccine delivery score of SCs across states. The MCA score was standardized between 0 and 100 and averaged using state household weighting for the figures. The infrastructure quality score ranged from 42 in Uttar Pradesh to 83 in Himachal Pradesh, and the vaccine service delivery score varied from 43 in Manipur to 100 in Delhi.

### 3.2 Probit regression results

Table 4 presents the results of the probit regression. In comparison with children whose SC’s infrastructure quality index score was in the lowest tercile, children whose SC’s score was in the highest tercile had significantly higher rates vaccination for all vaccines except for measles. Full vaccination and OTV were 1.19 (95% confidence interval [CI]: 1.02–1.38, p < 0.05) times and 1.2 (CI: 1.06–1.37, p < 0.05) times higher for children residing in districts with SC in highest tercile of infrastructure index relative to those in the first tercile, respectively. Children whose SC’s infrastructure quality index score was in the second tercile had significantly higher vaccination rates for DPT-1, DPT-3, BCG, and HepB relative to the lowest tercile. In comparison with the same reference group, children whose SC’s vaccine service delivery index score was in the third tercile had significantly higher rates of vaccination for measles (adjusted odds ratio [AOR] = 1.21, CI: 1.03–1.42, p < 0.05).

**Table 4:** Probit Regression Results of Immunization Outcomes and Health Facility Characteristics.

Model	1	2	3	4	5	6	7	8
Vaccine	Full	DPT-1	DPT-2	DPT-3	Measles	BCG	HepB	OTV
Vaccine availability score = 1								
2	1.11	1.04	1.01	1.02	1.13	1.02	1.1	1.08
	0.09	0.07	0.07	0.06	0.09	0.1	0.07	0.06
3	1.17+	1.11+	1.09	1.09	1.21*	1.13	1.13	1.07
	0.1	0.07	0.07	0.07	0.1	0.09	0.08	0.07
Infrastructure score = 1								
2	1.14	1.15*	1.12+	1.14*	1.11	1.27**	1.24**	1.12
	0.1	0.06	0.07	0.07	0.07	0.11	0.07	0.08
3	1.19*	1.17*	1.18**	1.20**	1.13	1.22*	1.34**	1.20**
	0.09	0.07	0.07	0.07	0.09	0.12	0.09	0.08
Region (Central = 1)								
East	1.60**	1.1	1.27*	1.42**	1.37**	1.39**	0.95	1.50**
	0.19	0.11	0.13	0.16	0.12	0.16	0.08	0.15
North	0.93	0.75**	0.83+	0.85	0.78**	0.68**	0.61**	1.05
	0.11	0.07	0.08	0.1	0.07	0.08	0.05	0.11
Northeast	0.96	1.02	1.06	1.07	0.78+	0.77	0.50**	1.42*
	0.17	0.17	0.17	0.16	0.12	0.2	0.11	0.22
South	1.25	1.44*	1.48**	1.45*	0.92	1.35	1.74**	1.13
	0.24	0.21	0.21	0.22	0.16	0.25	0.28	0.15
West	1.12	0.82	0.89	0.99	0.84+	0.67**	0.77*	1.08
	0.13	0.1	0.1	0.11	0.08	0.1	0.09	0.12
Age of Mother	1	1	1	1	1	1	1	1.01
	0	0	0	0	0.01	0.01	0	0
Sex (Male = 1)								
Female	0.99	1	1.03	1	0.98	1.09*	1.02	0.97
	0.04	0.04	0.03	0.02	0.03	0.04	0.04	0.03
Locality (Rural = 1)								
Urban	1.22+	1.06	1.07	1.13	1.09	1.02	0.96	1.08
	0.13	0.1	0.09	0.12	0.11	0.16	0.09	0.08
Age of Child	1	1.01**	1.01**	1.01**	1.02**	1.01**	1.01**	1.03**
	0	0	0	0	0	0	0	0
Wealth Quintile (1 = 1)								
2	1.22**	1.19**	1.18**	1.14**	1.20**	1.11+	1.16**	1.21**
	0.06	0.06	0.06	0.05	0.05	0.07	0.05	0.06
3	1.19**	1.16*	1.19**	1.12**	1.18**	1.27**	1.17*	1.15*
	0.08	0.07	0.07	0.05	0.07	0.08	0.07	0.07
4	1.23*	1.30**	1.32**	1.24**	1.26**	1.47**	1.35**	1.22**
	0.1	0.11	0.1	0.09	0.1	0.14	0.11	0.08
5	1.54**	1.33**	1.39**	1.34**	1.53**	1.48**	1.40**	1.35**
	0.15	0.11	0.12	0.09	0.14	0.16	0.12	0.09
Religion (Hindu = 1)								
Muslim	0.83*	0.84*	0.82*	0.9	0.78**	0.85	0.84*	0.83**
	0.06	0.07	0.07	0.07	0.06	0.11	0.06	0.05
Christian	0.76*	0.79+	0.74**	0.84	0.91	0.64*	0.85	0.84*
	0.09	0.1	0.08	0.09	0.11	0.13	0.13	0.07
Sikh	1.62*	1.44*	1.23	1.43+	1.58+	1.09	1.54*	1.20+
	0.35	0.25	0.24	0.3	0.41	0.18	0.27	0.11
Other	0.84	1.19	1.01	0.9	0.82	0.82	0.59*	0.9
	0.13	0.21	0.2	0.12	0.17	0.16	0.14	0.13
Caste (General = 1)								
Scheduled tribe	0.84	0.88	0.92	0.82*	1.11	1.24+	1.01	0.80*
	0.09	0.09	0.08	0.07	0.11	0.16	0.09	0.07
Other backward caste	1.01	1.03	1	0.98	0.99	1.06	1.04	0.95
	0.08	0.09	0.06	0.06	0.08	0.14	0.08	0.05
Scheduled caste	0.97	1.09	0.98	0.98	0.98	1.13+	1.08	0.92
	0.06	0.07	0.06	0.06	0.09	0.08	0.06	0.05
Education (No schooling = 1)								
Primary or lower	1.15**	1.20**	1.20**	1.14**	1.16**	1.17**	1.23**	1.10*
	0.06	0.06	0.06	0.05	0.06	0.07	0.07	0.05
Middle to Secondary	1.22**	1.28**	1.27**	1.22**	1.13**	1.25**	1.12*	1.16**
	0.07	0.07	0.06	0.06	0.05	0.08	0.06	0.06
Graduate	1.17	1.67**	1.43**	1.26**	1.20+	1.47*	1.26*	1.11
	0.13	0.15	0.13	0.1	0.12	0.23	0.13	0.09
Household size (<5 = 1)								
> 5	0.93+	0.99	1.01	1.01	0.97	0.94	0.95	0.96
	0.04	0.04	0.04	0.03	0.05	0.05	0.04	0.03
Institutional Delivery (Institutional = 1)								
Non-Institutional	0.80**	0.77**	0.78**	0.78**	0.73**	0.62**	0.57**	0.84**
	0.05	0.04	0.04	0.04	0.04	0.06	0.04	0.05
Distance to vaccination center (<15 min = 1)								
15 to 30 min	0.86**	0.92+	0.92*	0.92*	0.85**	0.98	0.99	0.89**
	0.05	0.04	0.03	0.03	0.05	0.05	0.04	0.04
> 30 min	0.77**	0.71**	0.78**	0.85**	0.78**	0.87+	0.96	0.86**
	0.06	0.04	0.05	0.05	0.06	0.07	0.06	0.04
Observations	11,461	22,104	21,333	20,543	15,179	22,749	22,749	20,613
Pseudo R^2^	0.063	0.111	0.114	0.094	0.098	0.156	0.141	0.269
P-value for c2	0	0	0	0	0	0	0	0

Note: *Full =* 1 dose BCG and measles, 3 doses of DPT and polio*; HepB* = Hepatitis B given at birth, *DPT* = Diphtheria, Pertussis, Tetanus, *BCG* = Bacillus Calmette–Guérin; *OTV* = On-time vaccination – considers timely vaccination of DPT and full immunization. Standard errors are below coefficients. + p < 0.1, *p < 0.05, **p < 0.01.

Regarding other covariates, wealth quintile of household and maternal education level showed a significantly positive association with child vaccination outcomes. Non-institutional delivery of child and greater distance to vaccination site of household were significantly negatively associated with vaccination outcomes. Children from Muslim and Christian households had significantly lower levels of vaccination relative to Hindu households.

### 3.3 Sensitivity analysis

Analysis with alternative definitions of the OTV variable, where timely receipt of all eligible DPT vaccines and full immunization was evaluated instead of the last eligible vaccine, found infrastructure quality index continued to be significantly positively associated with these alternative definitions of OTV — with AORs of 1.19 (95% CI: 1.02 – 1.38) and 1.11 (95% CI: 1.05–1.17), respectively. Across the three INCHIS rounds, 37% of households did not have a verified vaccination card for the child and vaccination outcomes were reported by the mother or caregiver. In additional analyses, we excluded these observations and found that the infrastructure quality index was still significantly associated with DPT-2, DPT-3, BCG, HepB, and OTV vaccine outcomes. However, infrastructure quality index was not significantly associated with full vaccination and DPT-1 vaccination, and the vaccine service delivery index was not significantly associated with measles vaccination anymore.

### 3.4 Fairlie decomposition results

Table 5 shows the results of the Fairlie decomposition analysis for the poorest households (those in wealth quintile 1, or WQ1) relative to other households (WQ2-WQ5) for full immunization and OTV. The difference in full vaccination and OTV rates between WQ1 and WQ2-WQ5 was 13% for full immunization and 5% for OTV. The unexplained gap — gap not explained by differences in the distribution of included covariates — between WQ1 and WQ2-WQ5 households was 56% and 29% for full immunization and OTV, respectively. The unexplained gap is due to structural differences in the returns to covariates across wealth groups.

**Table 5:** Decomposition of rural–urban gap in vaccination outcomes.

Covariates	Full	OTV
Region	0.0080+	−0.0071*
	0.0047	0.0032
Locality	0.0074**	0.0038*
	0.0026	0.0019
Religion	0.0009	0.0012+
	0.0011	0.0007
Caste	0.0029	0.0034+
	0.0027	0.0019
Age of Mother	−0.0039+	−0.0022+
	0.0022	0.0012
Sex	0	0
	0.0002	0.0001
Age of Child	0	−0.0177**
	0.0001	0.0004
Distance to sub-center	−0.0018*	−0.0005
	0.0007	0.0003
Maternal Education	0.0295**	0.0178**
	0.0066	0.005
Household Size	−0.0002	0.0001
	0.0002	0.0001
Institutional Delivery	0.0102*	0.0068*
	0.0044	0.0031
Infrastructure score	0.0073*	0.0077**
	0.0033	0.0022
Vaccine availability score	−0.0008	0.0004
	0.0021	0.0012
Total Gap	0.13	0.07
Explained Gap	0.059571	0.01
Explained Gap (%)	44	21
Sample size	12,793	23,000

Note: *Full =* 1 dose BCG and measles, 3 doses of DPT and polio*; OTV*: On-time vaccination – considers timely vaccination of DPT and full immunization. Standard errors are below coefficients. + p < 0.1, *p < 0.05, **p < 0.01.

The infrastructure quality index score of household’s SC contributed to a widening gap in full immunization and OTV rates between WQ1 and WQ2-WQ5 households, 5% and 11% of the total gap, respectively. For full immunization and OTV, differences in locality (urban vs. rural residence), institutional delivery rates, and maternal education levels, contributed to a wider gap between WQ1 and WQ2-WQ5 households. Differences in maternal education between rich and poor households contributed most to explaining vaccination differences, 22% of the total gap for full immunization and 25% for OTV. Differences in distance to vaccination site contributed to a decreased gap (1%) in full vaccination outcomes and age of child contributed to a reduction in gap in OTV between WQ1 and WQ2-WQ5 households.

## 4 Discussion

Missed child vaccinations in India continue to cause large burdens of preventable mortality and morbidity. In 2019–20, 76% of Indian children of age 12–23 months were fully vaccinated; state-wise, full vaccination rates ranged from 58% in Nagaland to 91% in Odisha [13]. Sub-national differences in vaccination rates contribute to higher child mortality in some states – in 2015, there were 10 deaths per 1,000 under-5 children in the Southern states, as compared with 40 deaths per 1,000 under-5 children in Northeastern states [1]. It is critical to understand the modifiable drivers of under-vaccination in India to decrease premature mortality.

While parents often report poor healthcare facility quality as a reason for not vaccinating their children, the association of health facility quality and vaccination remains largely unquantified in LMICs. Past studies have focused on access to care — availability of health facility or health workers — whereas the quality of care is known to be a more appropriate indicator of health outcomes [21], [22]. For example, a recent study in India looked at the proximal availability of a health facility and health care workers and found no effect on DPT vaccination dropout rates [16]. Another study in rural India looked at household proximity to a hospital and found a positive effect on vaccination rates but no effects of availability of community health workers on vaccination rates [17]. A third study focused on the slums of Agra, India, and found availability of health facility near households was positively associated with vaccination coverage [18]. In West Bengal, authors found that the availability of health workers and equipment at SCs was positively associated with month-specific vaccine coverage, while they found no effect of recent visit of supervisor to SC and the proportion of auxiliary nurses and midwives with immunization training in the SC [19]. In Burkina Faso, a study found no association between physical and human resources availability in health facilities and the community’s vaccination coverage [20]. Our findings move beyond these indicators of access and show that indicators of quality of health facilities are associated positively with vaccination outcomes.

A study similar to ours conducted in Pakistan found no association between district level indicators of healthcare staff availability and their knowledge, budget, and equipment, and child vaccination rates [8]. While our analysis is at the household level, the Pakistan study used aggregated district level data which may have omitted important variations at the household or individual level. Another major difference between our studies is their use of individual facility indicators as independent variables (e.g., syringe availability, budget, and number of staff visits made), whereas we used a composite index to measure infrastructure quality and equipment and vaccine availability.

An additional contribution of our study is examining the interplay of facility quality and household standard of living using a decomposition technique. While public health resources are meant to aid the most underserved communities which cannot access private sector healthcare, we found that lower income households had access to lower quality health facilities than higher income households, demonstrating a failure in the equitable distribution of public health resources. We did however find that the average distance to the closest health facility was smaller for lower income households and it decreased the gap in vaccination between income groups.

Our results have several policy implications. Health infrastructure quality in our study — measured by components of physical infrastructure (availability of regular power source, washroom, and building materials), observational assessment of the building by the surveyor, and ASHA education —was a proxy for overall facility resource availability and was positively associated with vaccination outcomes. The estimated associations were similar in magnitude to those of wealth quintile and maternal education indicators. This suggests that health infrastructure, including well-trained health workers, equipment, and facilities, could improve vaccination outcomes at the same rate as improvements in standard of living or maternal schooling [2]. In addition, vaccine service delivery quality – as measured by the availability of vaccines and associated medical supplies – may improve the coverage of measles vaccine more as compared with the physical infrastructure of SCs. This might be related to the timing of vaccine doses – in our analysis, the measles vaccine is the last vaccine (at age 9–12 months). One hypothesis may be that receipt of vaccines later in the series may depend more on the availability of vaccines and associated supplies instead of the general infrastructure of the SC as households have lesser contact with the health system as a child ages.

On the demand side, access to health facilities may play an important role in the household decision-making process related to child immunization. Over 24% of respondents gave one of the following as a reason for not vaccinating their children in INCHIS: not knowing benefits of vaccines, vaccination schedule, or distance to the vaccination site; and not having enough time to take child to vaccination site. Reasons directly related to health infrastructure for not vaccinating their child were vaccination site was too far (9%), vaccination site was unhygienic (3%), vaccine was not available (11%), and the ANM was not available (10%).

In our decomposition analysis, overall infrastructure quality contributed to an increasing gap in full immunization and OTV between rich and poor households. This suggests that richer households are serviced by SCs that have greater overall infrastructure and financing relative to SCs near poorest households. The distance to SC, which is associated positively with vaccination, was shorter for the poorest households on average and decreased the gap in vaccination. Therefore, while greater proximity of facilities to poorer households or a greater number of facilities existing in low-income districts contributes to decreasing vaccination disparities, there needs to be an increased investment in the infrastructure of these facilities. However, the UIP annual budget currently at $2 billion [47] is underfunded — estimates suggest annual budgetary shortfalls ranging from $7.9 to $50.2 million (INR 56 crore to INR 3,537 crore, 1 USD = INR 70.52) during 2013–2017 [48]. More recent estimates from a 2021 study suggest an additional $560 million would be required annually to increase child vaccination rates to 90% [49]. These shortfalls may increase as new vaccines are introduced (e.g. pneumococcal conjugate vaccine) and universalized, and as GAVI funding to the immunization program decreases annually post-2017 [48].

Travel time to vaccination site was negatively associated with vaccination status in our results. Therefore, construction of more SCs to ensure easy access for all populations should be considered. These can be supplemented with more outreach immunization sessions for hard to reach populations. Furthermore, to reduce travel time to vaccination sites investments in physical infrastructure such as paved roads and the availability of public transportation should be assessed, especially for rural areas [50]. The opportunity cost to travel to these vaccinations sites, particularly for the poor who lag in vaccination the most, may be too high if the site is prohibitively far [12]. These considerations should inform the location and timing of future vaccination centers to ensure equitable and timely access to vaccination in underserved communities. Additionally, we found large gaps in vaccination coverage across socio-economic subgroups in our study, consistent with past research [5], [29], [38]. Low-income and poorly educated households, as well as Muslim, Christian, and scheduled tribe households, continue to have worse vaccination outcomes, and vaccination efforts should target these groups.

Our study has important limitations. First, we used cross-sectional data which could be biased by selective program placement. For example, low-initial immunization coverage districts may have received additional resources to improve infrastructure to bolster vaccination rates, which could bias our coefficient on infrastructure quality index downwards. Conversely, richer communities with more political clout may have received more infrastructural resources — previous research have shown that public goods allocation in India can be often political [51], [52]. This would bias the estimated coefficients upwards as wealthier households tend to have higher levels of immunization. Future research should use longitudinal data to identify causal effects.

Second, we examined the quality of SC primarily through physical infrastructure quality and equipment availability, with the exception of ASHA education. Other measures of immunization delivery quality such as process quality can be included in future research [53]. Third, we matched each household with the sub-center in its own geographical cluster. This is the officially designated SC serving that household; however, it may be possible that households went to SC in other clusters if those centers were geographically closer to them. Use of data where the actual SC visited is identified may be desirable in future research.

Lasty, there may have been measurement error due to surveyor bias. While many of the facility quality indicators were based on objective measures (e.g., availability of vaccines), some indicators were based on the surveyor’s objective assessment of the state of the facility (e.g., cleanliness of the facility). However, potential inconsistencies across interviewers should have been minimized by training —all surveyors were presented with extensive instruction on the administration of the questionnaire and a training manual to specifically ensure uniformity across surveyor methods [23].

## 5 Conclusion

Immunization is a cost-effective tool for decreasing the high child mortality burden in India and other LMICs. This paper provides recent data on immunization outcomes and an analysis of the socio-economic and demographic groups that remain most vulnerable to being unvaccinated. It also adds to the limited research on the association of immunization outcomes with health infrastructure quality, showing that health facility quality could be an important determinant of immunization and the distribution of resources for health facilities can be better targeted towards more vulnerable groups.

## 6 Role of funding source

This work was supported, in whole, by the Bill & Melinda Gates Foundation [OPP1183738]. Under the grant conditions of the Foundation, a Creative Commons Attribution 4.0 Generic License has already been assigned to the Author Accepted Manuscript version that might arise from this submission. The funders had no role in study design, data collection and analysis, decision to publish, or preparation of the manuscript.

## Author contributions

All authors designed the study. AS conducted the analysis and drafted the initial manuscript. All authors interpreted the findings, and critically evaluated and edited the manuscript. All authors approved the final draft for publication.

## Declaration of Competing Interest

The authors declare that they have no known competing financial interests or personal relationships that could have appeared to influence the work reported in this paper.
